# Discussions and Misinformation About Electronic Nicotine Delivery Systems and COVID-19: Qualitative Analysis of Twitter Content

**DOI:** 10.2196/26335

**Published:** 2022-04-13

**Authors:** Jaime E Sidani, Beth Hoffman, Jason B Colditz, Riley Wolynn, Lily Hsiao, Kar-Hai Chu, Jason J Rose, Ariel Shensa, Esa Davis, Brian Primack

**Affiliations:** 1 Center for Social Dynamics and Community Health Department of Behavioral and Community Health Sciences University of Pittsburgh School of Public Health Pittsburgh, PA United States; 2 Division of General Internal Medicine University of Pittsburgh School of Medicine Pittsburgh, PA United States; 3 Kenneth P Dietrich School of Arts & Sciences University of Pittsburgh Pittsburgh, PA United States; 4 Division of Pulmonary, Allergy and Critical Care Medicine University of Pittsburgh School of Medicine Pittsburgh, PA United States; 5 Department of Health Administration and Public Health John G Rangos Sr School of Health Sciences Duquesne University Pittsburgh, PA United States; 6 College of Education and Health Professions University of Arkansas Fayetteville, AZ United States

**Keywords:** COVID-19, coronavirus, e-cigarette, electronic nicotine delivery systems, Twitter, social media, misinformation, discussion, public health, communication, concern, severity, conspiracy

## Abstract

**Background:**

Misinformation and conspiracy theories related to COVID-19 and electronic nicotine delivery systems (ENDS) are increasing. Some of this may stem from early reports suggesting a lower risk of severe COVID-19 in nicotine users. Additionally, a common conspiracy is that the e-cigarette or vaping product use–associated lung injury (EVALI) outbreak of 2019 was actually an early presentation of COVID-19. This may have important public health ramifications for both COVID-19 control and ENDS use.

**Objective:**

Twitter is an ideal tool for analyzing real-time public discussions related to both ENDS and COVID-19. This study seeks to collect and classify Twitter messages (“tweets”) related to ENDS and COVID-19 to inform public health messaging.

**Methods:**

Approximately 2.1 million tweets matching ENDS-related keywords were collected from March 1, 2020, through June 30, 2020, and were then filtered for COVID-19–related keywords, resulting in 67,321 original tweets. A 5% (n=3366) subsample was obtained for human coding using a systematically developed codebook. Tweets were coded for relevance to the topic and four overarching categories.

**Results:**

A total of 1930 (57.3%) tweets were coded as relevant to the research topic. Half (n=1008, 52.2%) of these discussed a perceived association between ENDS use and COVID-19 susceptibility or severity, with 42.4% (n=818) suggesting that ENDS use is associated with worse COVID-19 symptoms. One-quarter (n=479, 24.8%) of tweets discussed the perceived similarity/dissimilarity of COVID-19 and EVALI, and 13.8% (n=266) discussed ENDS use behavior. Misinformation and conspiracy theories were present throughout all coding categories.

**Conclusions:**

Discussions about ENDS use and COVID-19 on Twitter frequently highlight concerns about the susceptibility and severity of COVID-19 for ENDS users; however, many contain misinformation and conspiracy theories. Public health messaging should capitalize on these concerns and amplify accurate Twitter messaging.

## Introduction

The COVID-19 pandemic has spread rapidly, with over 490 million confirmed cases and over 6.1 million confirmed deaths worldwide at the time of this writing [1]. COVID-19, which can lead to acute respiratory distress syndrome, may be particularly dangerous for nicotine and tobacco users [2]. Emerging evidence suggests an association between the use of electronic nicotine delivery systems (ENDS) and greater incidence of COVID-19 susceptibility and severity, testing, and diagnosis—particularly among US adolescents and young adults [3]. This is concerning considering the increase in the use of these products. Worldwide, approximately 35 million individuals reported ENDS use in 2015, and this number is expected to increase as refillable and disposable ENDS become more popular [4].

The research on the potential associations between tobacco and nicotine use and COVID-19 risk has been mixed. Some published research has indicated that self-reported COVID-19 infection is greater among current cigarette smokers and former smokers compared to nonsmokers [5], that cigarette smoking is associated with higher odds of COVID-19 progression [6], and that ENDS use is associated with increased risk of COVID-19 infection [3]. However, a series of preprints suggesting an inverse relationship between tobacco and nicotine use and COVID-19 risk have also been released, some with substantial reach. For example, a preprint suggesting that cigarette smoking decreases the risk of COVID-19 infection by half was viewed over 56,000 times and has been tweeted 200 times at the time of this writing [7]. Likewise, a preprint suggesting that current cigarette smoking was inversely correlated with COVID-19 mortality has been viewed over 14,000 times [8]. A study using Twitter data found that sentiment toward cigarette smoking and ENDS use became more positive after the release of these preprints and non–peer-reviewed publications, suggesting that tobacco and nicotine users may be at less risk from COVID-19 infection and progression [9].

Likewise, research on the impact of COVID-19 on ENDS use has been mixed. A survey of a small convenience sample of US adult dual cigarette and ENDS users found that approximately one-quarter of participants attempted to reduce their tobacco and nicotine use during the pandemic [10]. Results from a five-country survey, which included the United States, also found an increase in quit attempts due to the pandemic; however, this study also showed little change in actual consumption of tobacco and nicotine products during COVID-19 lockdowns [11]. A qualitative study of ENDS users found that limited availability of ENDS products during lockdowns prompted them to turn to readily available cigarettes [12].

The COVID-19 pandemic has been accompanied by an “infodemic” in which a substantial amount of information has been spreading both online and offline [13]. In particular, misinformation about COVID-19 has been spreading on social media throughout the duration of the pandemic [14,15]. Misinformation about COVID-19 on Twitter has been found to spread virally within a matter of days, often fueling conspiracy theories [14]. Twitter is an ideal platform with which to conduct research on public opinion, conversations, and misinformation related to current health topics, including COVID-19 and ENDS. Most Twitter users maintain public profiles from which data can be obtained using Twitter’s Public Streams Application Programming Interface in real time, advancing itself as a tool for “infoveillance” [16,17]. Recently, Twitter data has been used to conduct preliminary work related to discussions around COVID-19 and ENDS, with the authors calling for a more systematic, in-depth qualitative examination of Twitter messages (ie, tweets) related to ENDS use and COVID-19 [18]. Another study examining Twitter data found that individuals who tweeted about ENDS during the pandemic expressed more concern about COVID-19 deaths compared to those who did not tweet about ENDS [19], but an in-depth qualitative analysis into the content of these tweets was not conducted.

Therefore, the purpose of this study was to systematically collect tweets related to COVID-19 and ENDS during the height of the pandemic in the United States and qualitatively analyze them to classify user discussions related to perceived associations between ENDS use and COVID-19. Using a “social listening” approach on Twitter can lead to a better understanding of tobacco-related topics of current importance [20]. Additionally, a qualitative approach allows for an in-depth exploration of discussions and often results in rich data that can be triangulated with quantitative results for a more complete understanding of a phenomenon. This could inform public health messaging and interventions related to ENDS use and COVID-19 misinformation throughout the remainder of the pandemic as well as future investigations of other misinformation related to ENDS and other tobacco products.

## Methods

### Data Collection and Sampling

We used the open source real-time infoveillance of Twitter health messages (RITHM) framework [17] to collect approximately 2.1 million tweets matching ENDS-related keywords and hashtags (vape, vapes, vaper, vapers, vaping, vaped, e-cigarette, e-cigarettes, e-cig, e-cigs, ecig, ecigs, juul, juuls, juuling) over multiple time points from March 1, 2020, through June 30, 2020, as recommended by Lienemann et al [21]. Of these, approximately half (1 million) were original tweets and the other half (1.1 million) were “retweets” (ie, rebroadcasts of others’ content). We then identified tweets containing keywords and hashtags related to the virus SARS-CoV-2 and the disease it causes, COVID-19 (sarscov2, sars-cov-2, covid, covid-19, covid19, corona, coronavirus, the rona, miss rona), which included 67,321 original tweets and 204,603 retweets. We next obtained a random 5% (n=3366) subsample of original COVID-19–related tweets for human annotation. Previous research has demonstrated that this approach maintains generalizability of the subsample within the context of the full data set [17,22]. This study was approved by the University of Pittsburgh Human Subjects Protection Office.

### Codebook Development and Coding Procedures

Initial codebook development involved a separate pool of random tweets (ie, not from the 3366 primary tweets). Individual codes were developed through a hybrid process, using both the themes identified by previous research and an examination by two independent coders of the pool of random tweets [18]. Coders reviewed and annotated these tweets and discussed potential codes with the lead author. After two rounds of this process, an initial codebook containing code and subcode names, definitions, and examples was developed (Table 1).

After initial codebook development, the two coders were provided with a spreadsheet containing the tweet text and a link to each tweet online. The tweet text was initially coded for relevance, defined as discussing a perceived association between COVID-19 and ENDS (eg, “almost 40% of ppl in the U.S. hospitalized for # COVID19 are between 20 and 54. #Vaping may be driving the rise in this” and “I’m going to juul the rona away”). Tweets that discussed ENDS or COVID-19 but not a perceived association between the two were excluded (eg, “coronavirus fears lessening in China as vape production goes back up there”). Coders viewed all relevant tweets that remained publicly available at the time of coding on Twitter so that links to external content could be assessed. However, coders included the text from unavailable tweets to preserve the comprehensiveness of the original data.

Relevant tweets were then coded as to whether they referenced four overarching categories: discussions about associations between COVID-19 severity and ENDS use; discussions about COVID-19 and e-cigarette or vaping product use–associated lung injury (EVALI) symptom similarity; discussions about COVID-19 affecting ENDS use; and discussions about personal or proximate experiences (eg, referencing something the tweeter saw themself or something that happened to someone the tweeter knows). Additionally, substantial misinformation related to COVID-19 and ENDS was found during coding and tweets containing potential misinformation—defined as statements not supported by the current peer-reviewed literature or exaggerations of research findings or public health findings—were identified and tagged by coders. Tweets containing potential misinformation were analyzed by an experienced graduate-level coder and the first author as themes within the major coding categories. All codes and subcodes are described in Table 1.

Codes were not mutually exclusive. For example, a tweet that stated, “Coronavirus attacks the lungs so one of the most important things you can do is to quit smoking and vaping. I’m in day 5 – join me!” would be coded as discussions about the association between COVID-19 severity and ENDS use (subcode: perception that ENDS use is associated with worse COVID-19 symptoms), discussions about COVID-19 affecting ENDS use (subcode: quitting ENDS because of COVID-19), and discussions about personal or proximate experience. We coded both textual and visual (eg, pictures, videos, and emojis) content [17].

The iterative coding process involved double-coding 100 tweets by two independent, experienced Twitter coders that were guided by a senior-level coder. All disagreements were discussed with the senior-level coder and adjudicated with the lead author, after which the codebook was modified accordingly. Interrater reliability was assessed using Cohen κ [23], and it was decided a priori that values above 0.70 would be acceptable. After four rounds of this process, Cohen κ reached acceptable levels of reliability (ranged 0.70-1.00) [24]. The two coders then independently coded the remaining tweets in the data set.

**Table 1:** Definitions for categorical codes and example tweets.

Code and subcode^a^			Definition		Examples^b^
**Discussions about the association between ENDS^c^ use and COVID-19 susceptibility or severity**			Tweet mentions that ENDS use is associated with contracting COVID-19 or severity of symptoms		
	Perception that ENDS use causes COVID-19	Tweet mentions that ENDS use may be a cause of developing COVID-19		“Vaping may be a cause of coronavirus cases in young people, experts say.” “PSA: vaping is an effective way to spread Covid 19! The viral aerosol mist stays in the air, so lots of your friends can catch the virus.”	
	Perception that ENDS use is associated with worse COVID-19 symptoms	Tweet mentions that ENDS use may be linked to worse COVID-19 symptoms/outcomes		“People who vape are more likely to experience negative effects from COVID-19.” “That vaping nic eliquid makes the Covid worse! Why do you continue with this?? Until there is data to confirm, just STOP IT! So tired of this!”	
	Perception that ENDS use protects against COVID-19	Tweet mentions that ENDS use can protect users from COVID-19 or make COVID-19 symptoms less severe		“If you've ever had vape juice get in your mouth after you take a hit you're immune to the corona virus. I said what I said.” “Juuling and vaping makes you immune to COVID.”	
**Discussions about COVID-19 and EVALI^d^ symptom similarity**			The tweet discusses both EVALI and COVID-19		
	Perception that EVALI is COVID-19	Tweet mentions thinking that EVALI was actually COVID-19		“Or, we already had the virus and they called it EVALI. CTs of COVID pts and EVALI patients look very similar.” “No ENDS was ever linked to EVALI. EVALI was just COVID a year early.”	
	Perception that EVALI is not COVID-19	Tweet mentions that EVALI and COVID-19 are distinct diseases		“The first cases of EVALI were reported in April 2019, way before covid. I don’t think they are related, but I could see how vaping makes it worse.”	
	Quitting ENDS because of COVID-19	Tweet mentions quitting ENDS use because of COVID-19		“In the middle of the COVID pandemic of a respiratory disease, smokers and vapers, now is a great time to think abt quitting before the habit kills you.” “Vaping nicotine makes coronavirus worse! Why do you all keep vaping? Until there is more data, just STOP vaping! So exhausted by this!”	
	Switching from combustible cigarettes to ENDS because of COVID-19	Tweet mentions switching from smoking cigarettes to using ENDS because of COVID-19		“I have converted so many people from smoking to nicotine vaping during this time of COVID-19!! 18 people have now made a healthier decision to use a harm reduction tool that really works!” “There are so many people switching to e-cigs and ditching traditional cigarettes. Vaping could help lower the number of people admitted to hospital if they get affected by the coronavirus.”	
	Starting or continuing ENDS use because of COVID-19	Tweet mentions starting or continuing using ENDS because of COVID-19		“I’ve just started vaping again as a way to manage stress. I’d quit nicotine for six months up until today...I’m blaming it on the Covid effect.” “COVID really has me back on my high-school diet of juul pods and iced lattes.”	
**Discussions about personal or proximate experiences**			Tweet contains reference to something the tweeter saw themself or something that happened to someone the tweeter knows		
	Respiratory symptoms	The tweet mentions symptoms that could be from COVID-19 or ENDS use, and the tweeter is not sure which is the cause		“About once or twice a week I’ll wake up with congestion, and I’m like well, I’ve got covid. And then I’ll remember that I burn through like 2 juul pods a day easy.” “He and my friends thought we had something before covid but we’re all smokers and thought it was from vaping – who knows?”	

^a^Code derived from original codebook; subcode derived from content analysis discussions and adjudications.

^b^Examples are provided for subcodes. Proper names and expletives have been censored. Minor details of tweet content were changed to prevent reidentification of individual Twitter users via direct quotes.

^c^ENDS: electronic nicotine delivery system.

^d^EVALI: e-cigarette or vaping product use–associated lung injury.

### Content Analysis

Frequencies and percentages were calculated for each code. A thematic qualitative content analysis approach was used to inductively assess the tweets and refine thematic units within codes [25]. The thematic analysis approach is recognized as a highly flexible qualitative approach that provides a rich and detailed account of data, especially within large data sets [26]. Qualitative themes and quotes around quantitative findings were organized to contextualize associations between COVID-19 and ENDS. Quotes were deidentified, and unique quotes were slightly rephrased while preserving the original meaning of the statement to prevent identification of individual Twitter users [17].

## Results

Of 3366 human-coded tweets, 1930 (57.3%) were coded as relevant (ie, discussed a perceived association between COVID-19 and ENDS) and were included in the analysis (Table 2). A total of 1008 (52.2%) tweets discussed the perceived association between COVID-19 susceptibility or severity and ENDS use, with a plurality (n=818, 42.4%) suggesting that ENDS use is associated with worse COVID-19 symptoms. Overarching themes focused on how young people should be concerned about this association because they are more likely to use ENDS than older people and how ENDS use damages the lungs and weakens the immune system. Tweets containing these themes were a mixture of news headlines and personal opinions.

Some tweets (n=120, 6.2%) suggested that ENDS use protects individuals from COVID-19 infection and progression. One theme focused on the potential curative effect of ENDS, with references to early research suggesting the protective effect of nicotine (eg, “Doctors in France recognize the power of nicotine to fight COVID-19 virus. Nicotine & vaping may become a preventive treatment & cure for COVID-19”) and the components of ENDS that may cure those with COVID-19 (eg, “Vaping most likely kills COVID because of the propylene glycol content in it”). Other themes suggested that ENDS use protects users from infection and that there was no link between COVID-19 and ENDS (ie, neither protective nor harmful).

Fewer tweets (n=80, 4.2%) suggested that ENDS use actually is the cause of COVID-19, with themes focused on the possibility that the COVID-19 virus was in ENDS liquid (eg, “Remember that mysterious illness caused by vapes in January? A severe respiratory illness. Well the first e cigs came from Wuhan China. What if they put Covid in vape juice, causing the illness and the spread?”) and that secondhand vapor might be contributing to the spread of COVID-19 (eg, “Public Service Announcement: Vaping is an effective way to spread COVID-19! The viral aerosol mist stays in the air, and your friends and family can catch the virus. DON’T VAPE”).

A total of 479 (24.8%) tweets discussed the perceived similarity (or dissimilarity) of the symptoms of COVID-19 and EVALI. Of these, a greater number of tweets (n=424, 22%) suggested that COVID-19 and EVALI are actually the same disease, with overarching themes focused on government deception (eg, “America is the epicenter and origin of coronavirus, But Trump and American Government have cheated the world since vaping- pneumonia erupted in August 2019. The Covid-19 patient 0 is from Fort Detrick. #TrumpLiedPeopleDied”) and similarities of symptoms and medical imaging. A smaller number of tweets (n=57, 3%) focused on distinctions between COVID-19 and EVALI, with overarching themes mentioning how EVALI was not infectious (eg, “If it was true that the vaping deaths were coronavirus, you would see patient-to-healthcare-worker infections”) and differences in age groups affected (eg, “Why didn’t any old people get EVALI then? It was all young people who vape”).

**Table 2:** Frequencies of coding categories for relevant tweets (n=1930).

Code and subcode		Frequency, n (%^a^)
**Discussions about the association between ENDS^b^ use and COVID-19 susceptibility or severity**		1008 (52.2)
	Perception that ENDS use causes COVID-19	80 (4.2)
	Perception that ENDS use is associated with worse COVID-19 symptoms	818 (42.4)
	Perception that ENDS use protects against COVID-19	120 (6.2)
**Discussions about COVID-19 and EVALI^c^ symptom similarity**		479 (24.8)
	Perception that EVALI is COVID-19	424 (22.0)
	Perception that EVALI is not COVID-19	57 (3.0)
**Discussions about COVID-19 affecting ENDS product use**		266 (13.8)
	Quitting ENDS because of COVID-19	180 (9.3)
	Switching from combustible cigarettes to ENDS because of COVID-19	33 (1.7)
	Starting or continuing ENDS use because of COVID-19	59 (3.1)
**Discussions about personal or proximate experiences**		231 (12.0)
	Respiratory symptoms	40 (2.1)

^a^Row percentages may not equal 100 due to rounding.

^b^ENDS: electronic nicotine delivery system.

^c^EVALI: e-cigarette or vaping product use–associated lung injury.

Approximately 13.8% (n=266) of tweets discussed a potential relationship between ENDS use behavior and the pandemic. Of these, most mentioned quitting ENDS in response to the pandemic (n=180, 9.3%). The most prominent theme in this category was quitting ENDS because of its effects on respiratory health (eg, “This is an excellent reason to quit smoking and vaping...those habits decrease your lungs' ability to keep clean and fight off coronavirus infection. Do not make it easier to get sick or sicker”). Fewer (n=59, 3.1%) mentioned starting or continuing ENDS because of the pandemic (eg, “corona got me thinkin bout my health so i got a juul for in b/w cigs”) and the perceived health benefits of nicotine (eg, “YOU NEED TO VAPE. Nicotine users are at a lower risk of developing COVID-19 symptoms...”). Finally, 33 (1.7%) tweets mentioned switching from traditional cigarettes to ENDS, with all tweets in this coding category containing the theme of ENDS being a safer alternative to cigarette smoking.

Approximately 12% (n = 231) discussed a personal or proximate experience. Among users who mentioned ENDS use themselves, one theme focused on limiting the sharing of their ENDS because of COVID-19 (eg, “Because of COVID, no you cannot hit my vape”). A total of 40 (2.1%) tweets discussed respiratory symptoms that users believed could be due to either COVID-19 or ENDS use (eg, “Was it it the constant vaping that gave me a sinus infection or do I have the rona”). Another theme focused on possibly having COVID-19 in the past but attributed symptoms to ENDS use at the time. Additionally, some users expressed concern about COVID-19 for friends/family who use ENDS (eg, “I think there's something very serious we need to address regarding Covid-19 and young people, considering 90% of the people I know use e-cigarettes and vapes perpetually”) and relief about not using ENDS themselves in light of COVID-19.

## Discussion

In this study, approximately half of the tweets that discussed perceived associations between ENDS use and COVID-19 contained language suggesting the perception that ENDS may worsen COVID-19—specifically that the use of ENDS by predominantly younger individuals may increase risk of severe COVID-19 symptoms. This is consistent with recent research finding that, among those aged 13 to 24 years, current ENDS users and current ENDS/cigarette dual users are 5 and 6.8 times more likely to be diagnosed with COVID-19 compared to nonusers, respectively [3]. This is also consistent with research finding that ENDS may have adverse effects on the cardiovascular and respiratory systems, and increase risk for infections, especially when combined with traditional cigarettes [27]. Likewise, tweets from users indicating a desire to quit ENDS were consistent with research suggesting that approximately one-quarter of US adult tobacco users sought to reduce their use during the pandemic [10].

The themes emerging from this study, combined with previous research, suggest that focusing public health messaging on the potential for worse COVID-19–related health outcomes among ENDS users may resonate with those discussing this topic on social media. Specific strategies, such as magnifying accurate Twitter messages linking ENDS use with COVID-19 and disseminating messages via social media with clear actionable public health advice linked to credible sources may be important health communication tools moving forward [28].

Our findings were also consistent with others who have reported rapid spread of misinformation related to COVID-19 in general [29,30]. In our study, one apparent source of misinformation was the preponderance of preprints of COVID-19–related research that had not yet undergone peer review and were later contradicted [9,31]. For example, we found that unsubstantiated reports that tobacco and nicotine users were at less risk for COVID-19 complications were being cited by Twitter users as justification to begin or maintain ENDS use [7,8]. Development and dissemination of counter-messaging clarifying the evidence related to ENDS use and COVID-19 may be useful at curtailing this spread of misinformation [32].

Additionally, our analysis uncovered conspiracy theories related to the origins of the virus, its transmissibility, and potential treatments [29,33]. For example, multiple tweets suggested that COVID-19 and EVALI were in fact the same condition. Several other tweets suggested that China engineered the virus and transmitted it to the United States via ENDS. Prior to COVID-19, concerns about EVALI appeared to have contributed to declines in ENDS use among youth [34]. If youth begin to equate EVALI with COVID-19 but do not believe in the dangers of COVID-19 or are no longer concerned about a uniquely vaping-related condition, this trend may reverse. Because the symptoms of EVALI and COVID-19 are similar, it is suggested that clinicians assess ENDS use during all clinical encounters in which COVID-19 is suspected [35].

Several tweets suggested that propylene glycol may kill the virus, thus protecting ENDS users from infection, which relates to a popular misperception of the antiviral and antibacterial properties of propylene glycol gas [32]. Some of these individuals also suggested that regulations around ENDS, including flavor bans, were initiated by the US government in an attempt to hoard propylene glycol for use with COVID-19 treatment. While more research is needed to better understand the relationship between the online spread of conspiracy theories and ENDS use behavior, these findings emphasize the value of using social media to monitor current discourse about various public health crises [13]. The real-time nature of Twitter allows for the capture and analysis of health-related information, misinformation, and disinformation more quickly than traditional methods such as surveys. Additionally, the use of techniques such as social network analysis can help assess the reach and spread of these messages, which can allow for the development of targeted interventions to mitigate the sharing of mis- and disinformation.

Our study was limited in that these results are neither generalizable to non-Twitter users nor the general population. Twitter users tend to be younger and more educated than the general population [36], and the content in the analyzed tweets may reflect that. Moreover, while we endeavored to collect a random sample of tweets, collected tweets are not necessarily representative of all Twitter content on this topic. It is also a necessary limitation that the tweets in this study were coded and analyzed by human coders. However, a series of steps were taken to mitigate this concern. First, we used highly trained and experienced Twitter coders that were guided by a senior-level coder. Second, our codebook was systematically developed and contained specific definitions and examples to guide coders in their interpretations. Third, we conducted four rounds of double-coding until sufficient interrater reliability was reached. At each round, coders discussed inconsistent results with the senior-level coder. A final limitation is that no conclusions about an association between ENDS and COVID-19 can be made from this study. Instead, this study consisted of qualitative analyses of discussions about ENDS and COVID-19.

In conclusion, discussions about the perceived associations between ENDS and COVID-19 on Twitter are often conflicting. These conflicts reflect the lack of consistent health communication messaging, which may have facilitated the spread of speculation and misinformation. The results suggest the need for further research to investigate the spread of information and misinformation about ENDS use and COVID-19, especially on social media platforms. They also suggest potential targets for evidence-based clarifications public health providers can implement.

## Abbreviations

ENDS: electronic nicotine delivery system
EVALI: e-cigarette or vaping product use–associated lung injury
RITHM: real-time infoveillance of Twitter health messages
